# Internal anatomy of a fossilized embryonic stage of the Cambrian-Ordovician scalidophoran *Markuelia*

**DOI:** 10.1098/rsos.220115

**Published:** 2022-10-05

**Authors:** Xi-ping Dong, Baichuan Duan, Jianbo Liu, Philip C. J. Donoghue

**Affiliations:** ^1^ School of Earth and Space Science, Peking University, Beijing 100871, People's Republic of China; ^2^ Key Laboratory of Marine Geology and Metallogeny, First Institute of Oceanography, Ministry of Natural Resource, Qingdao 266061, People's Republic of China; ^3^ Bristol Palaeobiology Group, School of Earth Sciences, University of Bristol, Life Sciences Building, Tyndall Avenue, Bristol BS8 1TQ, UK

**Keywords:** preserved, internal, anatomy, fossilized, embryonic, stage

## Abstract

The Wangcun fossil Lagerstätte in Hunan, South China, has yielded hundreds of fossilized embryos of *Markuelia hunanensis* representing different developmental stages. Internal tissues have only rarely been observed, impeding further understanding of the soft tissue anatomy, phylogenetic affinity and evolutionary significance of *Markuelia*. In this study, we used synchrotron radiation X-ray tomographic microscopy (SRXTM) to study a new collection of fossil embryos from the Wangcun fossil Lagerstätte. We describe specimens exhibiting a spectrum of preservation states, the best of which preserves palisade structures underneath the cuticle of the head and tail, distinct from patterns of centripetal mineralization of the cuticle and centrifugal mineralization of hypha-like structures, seen elsewhere in this specimen and other fossils within the same assemblage. Our computed tomographic reconstruction of these mineralization phases preserves the gross morphology of (i) longitudinal structures associated with the tail spines, which we interpret as the proximal ends of longitudinal muscles, and (ii) a ring-shaped structure internal to the introvert, which we interpret as a ring-shaped brain, as anticipated of the cycloneuralian affinity of *Markuelia*. This is the first record of a fossilized nervous system in a scalidophoran, and the first instance in Orsten-style preservation, opening the potential for further such records within this widespread mode of high-fidelity three-dimensional preservation.

## 1. Introduction

Ecdysozoa comprise one of the most diverse and disparate clades among animals, including the eight phyla: Arthropoda, Onychophora, Tardigrada (which together compose Panarthropoda), Nematoda and Nematomorpha (which compose Nematoida), and Kinorhyncha, Loricifera and Priapulida (which compose Scalidophora). These include a number of key genome and developmental models, including the nematode *Caenorhabditis elegans* and the arthropod *Drosophila melanogaster*, from which many of the most fundamental insights into molecular genetic controls on animal development and evolution, have been obtained. Despite this, the nature of ancestral ecdysozoan bodyplans remains unclear as a consequence of uncertainty over the evolutionary relationships of living ecdysozoans, as well as of their extinct relatives. This is unfortunate since even small amounts of fossil information have been demonstrated to have a significant impact on our understanding of deep ecdysozoan evolution, such as with the discovery of *Markuelia*, a genus of extinct (early Cambrian to early Ordovician) scalidophoran worms known only from embryonic stages of development [1].

*Markuelia* is known from early cleavage stages [1–3], but it is more commonly known from late embryos that are sub-millimetric in scale, annulated, with a terminal mouth cone surrounded by radially arranged circlets of hollow scalids associated with an introvert, and a terminal anus with bilaterally arranged, morphologically differentiated tail spines [1,2,4,5]. Given its resemblance to living and fossil priapulid adults, it is not surprising that phylogenetic analyses resolve *Markuelia* as a total-group scalidophoran and a stem scalidophoran in particular [1,2,4,6–12]. *Markuelia* has been interpreted to exhibit direct development, and its comparatively large size relative to the otherwise largely meiofaunal indirect developing scalidophorans has been interpreted to reflect large size and direct development among ancestral scalidophorans and, perhaps, ecdysozoans more generally [1,2,4,13]. Nevertheless, knowledge of the anatomy of *Markuelia* is limited not only to embryonic developmental stages, but also largely to external anatomy. Tomographic analysis has revealed preservation of the digestive tract extending a couple of hundred microns rostrad of the anus [12], but preservation is otherwise limited to the cuticle [14]; trunk musculature has been reported by Cheng *et al*. [15], but the evidence is not altogether convincing.

From among a new collection of embryos of *Markuelia hunanensis* recovered from limestones from the upper Cambrian (Furongian) Bitiao Formation at Wangcun Section, Yongshun County, western Hunan Province, South China [16], we describe a specimen that preserves aspects of the internal anatomy in the anterior and posterior regions. These features were tentatively interpreted as musculature in a preliminary study [17] but further investigation reveals that structures in the anterior region are comparable in size, shape and position to the circumoral brain of crown scalidophorans, with posterior projections possibly indicating the presence of paired nerve cords as seen in Nematoda and Kinorhyncha; the posterior structure may represent a caudal ganglia or musculature. This is the first time that fossil nervous tissues have been recorded as preserved in three dimensions and in a calcium phosphate medium. The structures are compatible with the view that the circumoral brain is a primitive feature for scalidophorans and, indeed, ecdysozoans; the unpaired nerve cords of living scalidophorans may be derived from an ancestral condition in which nerve cords were paired, as evidenced by *Markuelia* and extant nematodes.

## 2. Methods

### 2.1. Sample preparation and scanning electron microscope analysis

The limestone samples were dissolved in dilute acetic acid following the protocol of Müller [18], and the insoluble residue was manually sorted with the aid of a binocular microscope. Specimens were imaged using an environmental scanning electron microscope (ESEM, Quanta 200F) at the School of Earth and Space Sciences, Peking University.

### 2.2. Synchrotron radiation X-ray tomographic microscopy analysis and three-dimensional reconstruction

Thirteen specimens were characterized tomographically using synchrotron radiation X-ray tomographic microscopy (SRXTM) at the X02DA TOMCAT beamline of the Swiss Light Source, Paul Scherrer Institute, Villigen, Switzerland. SRXTM data were obtained using a 20 µm LuAg:Ce scintillator, 20x objective lens (yielding 0.325 µm voxel resolution), at an energy level of 14 keV and an exposure time of 200 ms, as a series of 1501 equiangular projections while the sample is rotated through 180 degrees within the beam. Projections were post-processed and rearranged into flat- and dark-field-corrected sinograms, and reconstruction was performed on a 60-core Linux PC farm, using a highly optimized routine based on the Fourier transform method and a regridding procedure [19]. Slice data were analysed and manipulated using AVIZO 8.0 (ThermoFisher Scientific), and the illustrated figures were assembled using Adobe PHOTOSHOP CS. Figured specimens are deposited in the Geological Museum of Peking University (GMPKU), Beijing. Given that the X-rays from the synchrotron sources are monochromatic, differences in contrast in the resulting tomographic slices reflect the densities of the fossil materials they pass through [20].

## 3. Results

### 3.1. External anatomy

**Figure 1.**
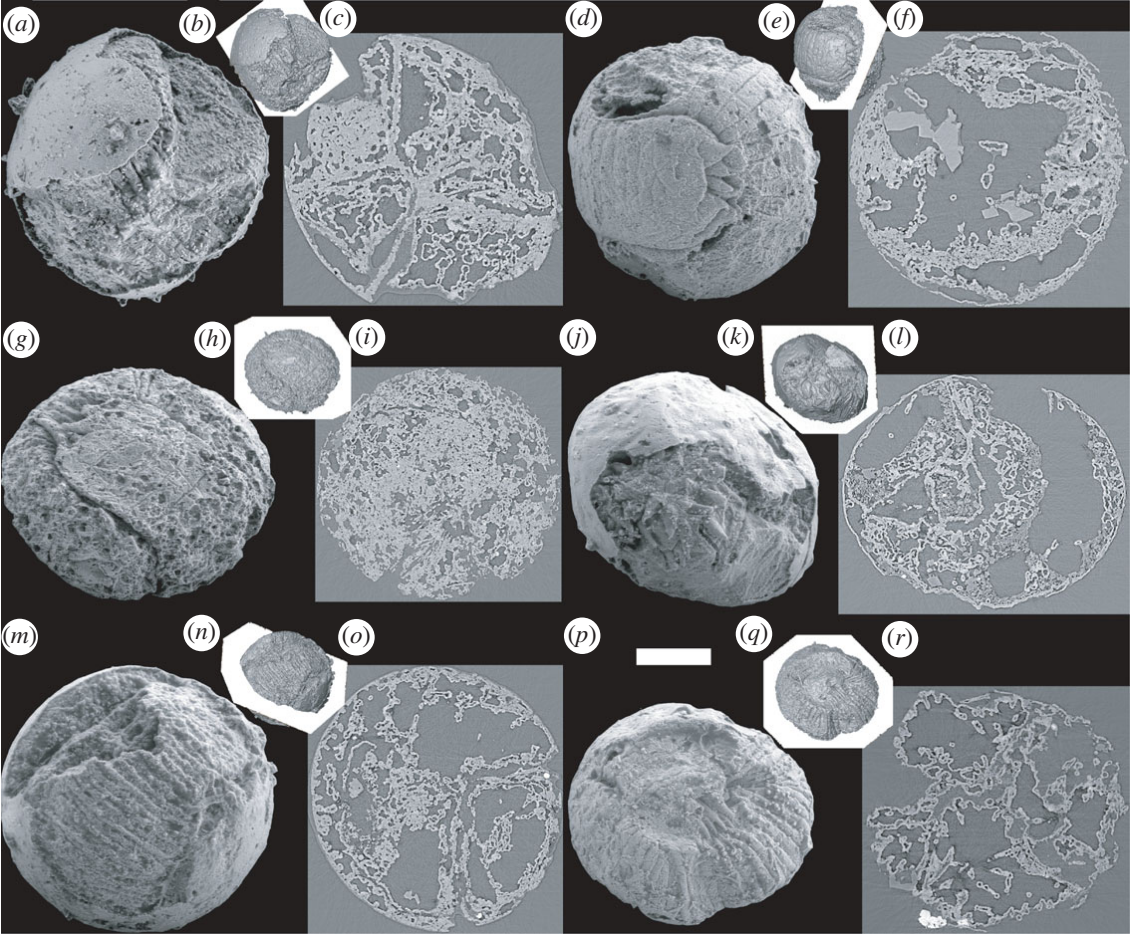
SEM images, surface images and orthoslices of six scanned fossil embryo specimens of *Markuelia hunanensis* Dong and Donoghue, 2004. (*a*) SEM image of GMPKU3140; (*b*) surface image of (*a*), oriented like (*a*), showing the relative position of the orthoslice; (*c*) orthoslice of (*a*). (*d*) SEM image of GMPKU3141; (*e*) surface image of (*d*), oriented like (*d*), showing the relative position of the orthoslice; (*f*) orthoslice of (*d*), showing euhedral space-filling calcite crystals. (*g*) SEM image of GMPKU3142; (*h*) surface image of (*g*), oriented like (*g*), showing the relative position of the orthoslice; (*i*) orthoslice of (*g*). (*j*) SEM image of GMPKU2388; (*k*) surface image of (*j*), oriented like (*j*), showing the relative position of the orthoslice; (*l*) orthoslice of (*j*). (*m*) SEM image of GMPKU3143; (*n*) surface image of (*m*), oriented like (*m*), showing the relative position of the orthoslice; (*o*) orthoslice of (*m*). (*p*) SEM image of GMPKU3144; (*q*) surface image of (*p*), oriented like (*p*), showing the relative position of the orthoslice; (*r*) orthoslice of (*p*). Relative scale bar, 103 µm (*a*), 260 µm (*b*), 103 µm (*c*), 104 µm (*d*), 294 µm (*e*), 104 µm (*f*), 110 µm (*g*), 315 µm (*h*), 110 µm (*i*), 115 µm (*j*), 302 µm (*k*), 115 µm (*l*), 103 µm (*m*), 295 µm (*n*), 103 µm (*o*), 125 µm (*p*), 297 µm (i), 125 µm (*r*).

The new collection of specimens provides an effective corroboration of previous descriptions of the external anatomy of *Markuelia hunanensis* based on a smaller database. All of the specimens represent comparatively late stages of development that are either spheroidal or strongly circular in outline with two approximately planar and parallel sides, joined by an open convex rim (figure 1). These subsequent laterally compressed specimens presumably reflect the very latest stages of embryonic development since their fertilization envelope is effectively deformed by the large size of the enclosed embryo. The envelopes are typically 400 to 500 µm in diameter, and the embryo is visible either through an intact envelope, or else where the envelope is broken (figure 1*a–c,j–o*); some specimens lack an envelope altogether (figure 1*d–i,p–r*), but we interpret this to have been lost biostratinomically, since the organism invariably maintains its characteristic embryonic enrolment into an S- or Z-shaped spheroidal loop, with head and tail juxtaposed in opposing orientations (figure 1*d,e,g,h*). In all specimens, the trunk is annulated (figure 1), and where preserved, the tail is characterized by three bilaterally arranged pairs of spines, two pairs of which are recurved and nested relative to a pair of central and approximately straight spines (figure 1*d,e,g,h,p,q*). The head is least commonly preserved but a number of specimens in our collection preserve a terminal mouth surrounded by 25 scalids arranged in a series of circlets (figure 1*j,k*) in a manner compatible with previous descriptions [1,12].

### 3.2. Internal anatomy

The majority of specimens preserve shallow internal divisions corresponding to the furrows defining the trunk annulae externally. Otherwise, internally, the embryos are dominated by anastomosing filaments that are enveloped by layers of diagenetic calcium phosphate mineralization. Most of the specimens are otherwise hollow but some preserve euhedral space-filling calcite crystals (due to incomplete acetic acid digestion); given the specimens are recovered by acetic acid dissolution of the limestone cement, it is likely that all of the specimens were originally void-filled by calcite (figure 1*c,f,i,o,r*).

**Figure 2.**
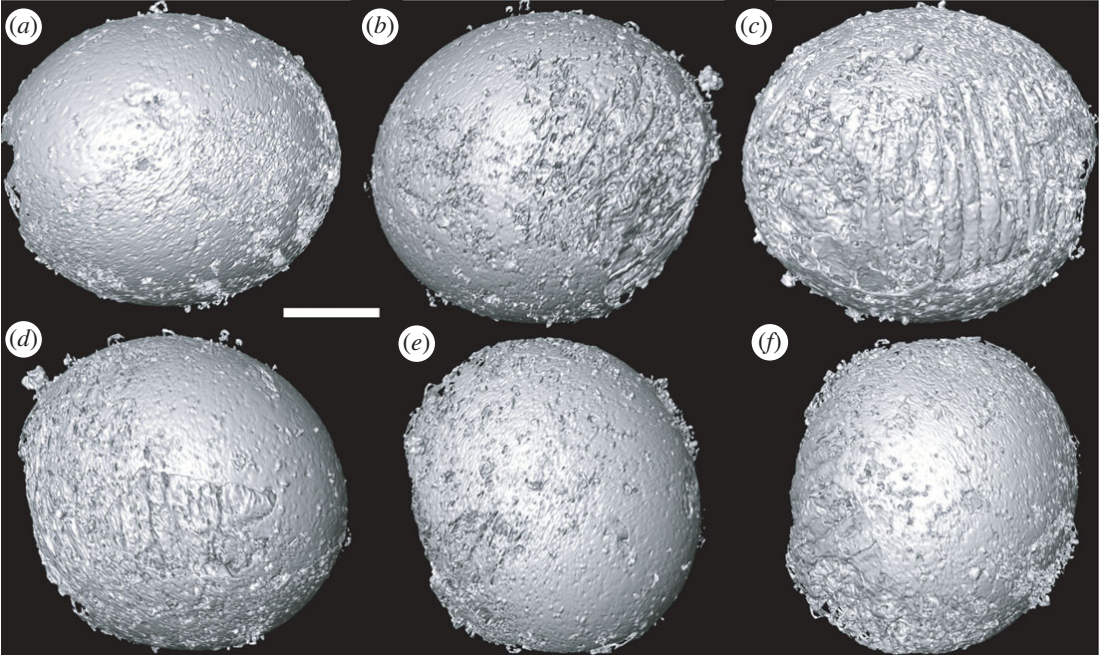
Tomographic surface models of gross morphology of *Markuelia hunanensis* in embryo stage (GMPKU3139). (*a*) Front, (*b*) left, (*c*) back, (*d*) right, (*e*) up and (*f*) down view of the specimen. Scale bar represents 100 µm.

**Figure 3.**
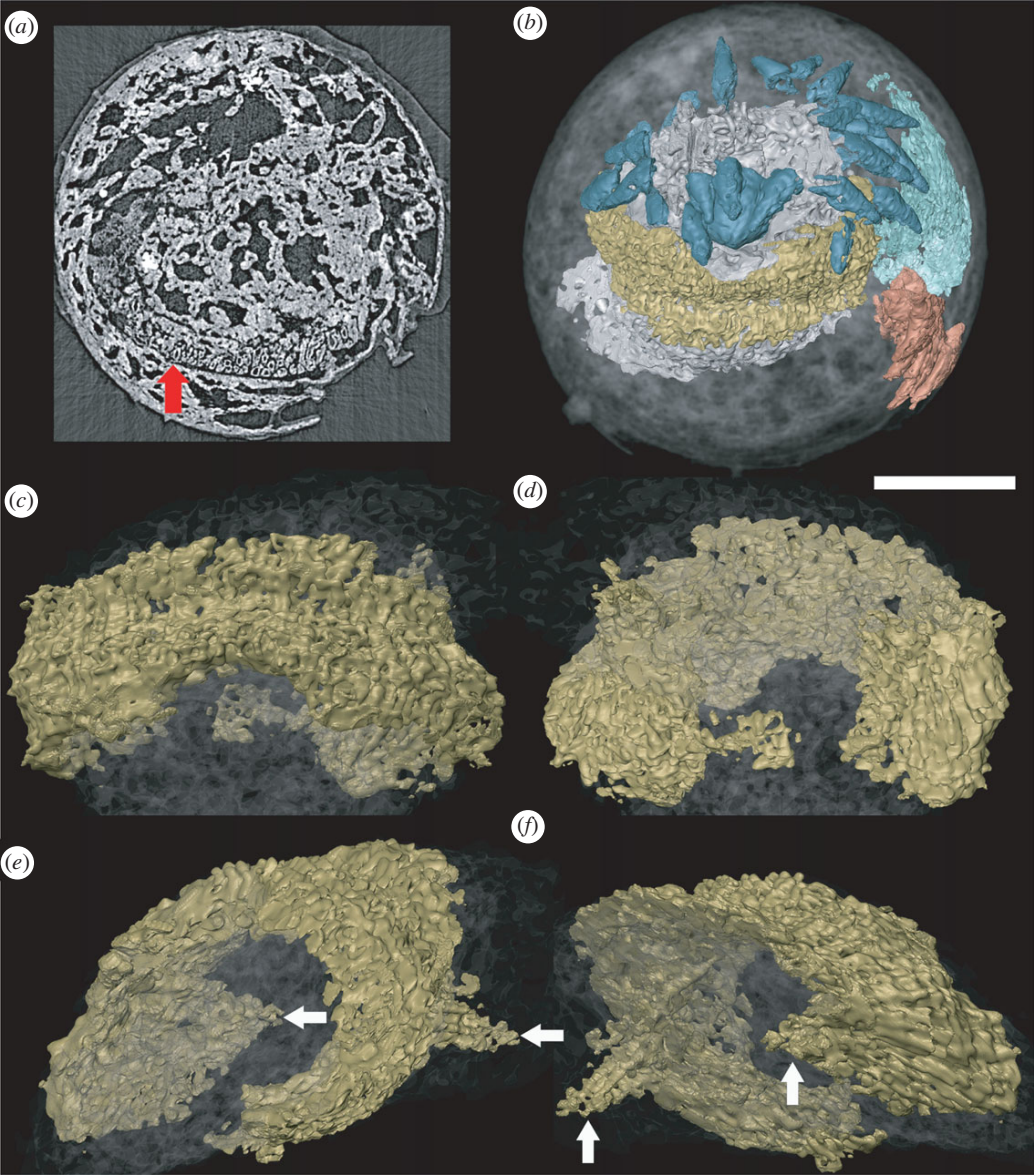
Reconstruction of the head of *Markuelia hunanensis*, showing the position and morphology of the palisade structure (GMPKU3139). (*a*) Palisade structure in the slice (arrowed). (*b*) General structure of the head and tail, showing the position of oral scalids (blue), pharynx (grey), brain (yellow), tail spines (orange) and posterior palisade structure (green). (*c*) Dorsal view, (*d*) ventral view, (*e*) left view and (*f*) right view of the oral palisade structure. Arrows in (*e*) and (*f*) point to paired projections. Scale bar represents 100 µm (*a*,*b*); 60 µm (*c*,*d*,*e*,*f*).

**Figure 4.**
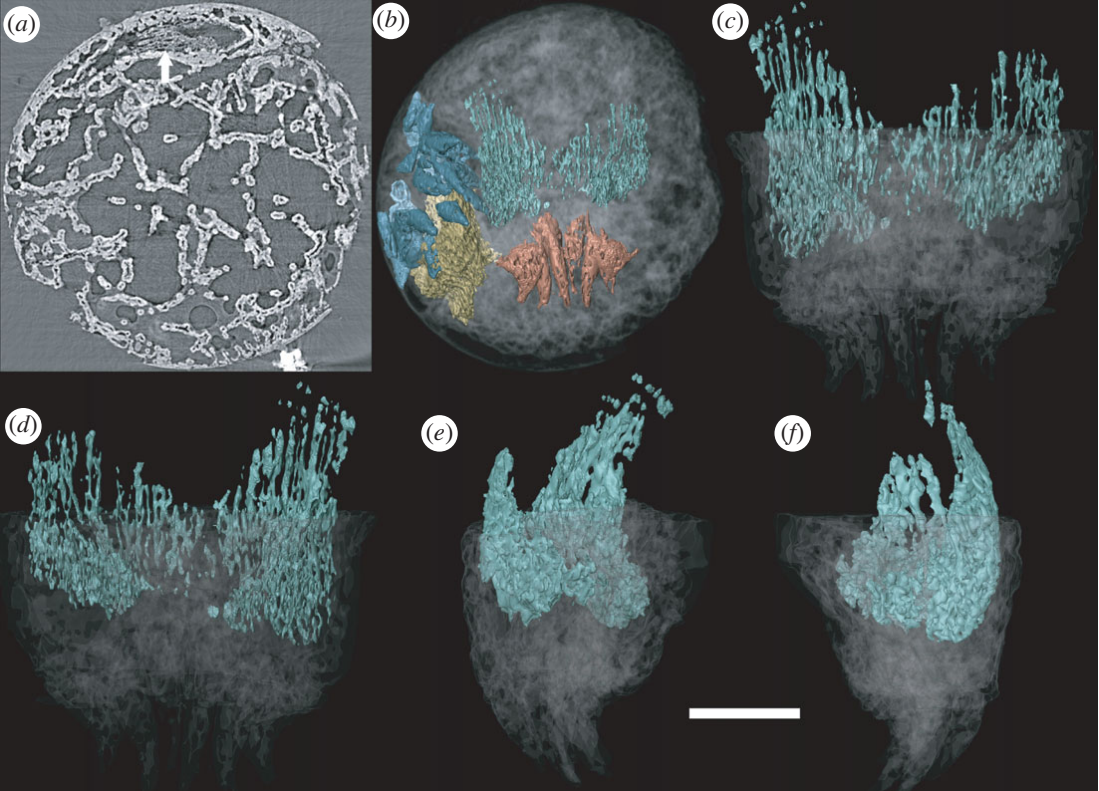
Reconstruction of the tail of *Markuelia hunanensis*, showing the position and morphology of the palisade structure (GMPKU3139). (*a*) Palisade structure in the slice (arrowed). (*b*) General structure of the tail, showing the position of tail spines (orange) and posterior palisade structure (green). (*c*) Dorsal view, (*d*) ventral view, (*e*) right view and (*f*) left view of the posterior palisade structure. Scale bar represents 100 µm (*a*,*b*); 54 µm (*c,d,e,f*).

A unique specimen preserves additional structures that we interpret to reflect distinct aspects of internal anatomy (figures 2 and 3). These structures are manifest as regions with a distinct mineralization fabric composed of elongate hollow trabecular elements (palisades) that are approximately 10 µm in diameter and body aligned approximately with the anterior–posterior axis of the organism (figures 3 and 4). The trabecular elements are conjoined by cross walls and comprise two larger structures (one anterior and one posterior), each with a regular overall morphological structure that is distinct from the shape of the space that they occupy, precluding interpretation as void-filling mouldic morphology. This contrasts strongly with the disordered arrangement of calcium phosphate mineralized filaments that otherwise occupy the void space (figures 3*a* and 4*a*).

**Figure 5.**
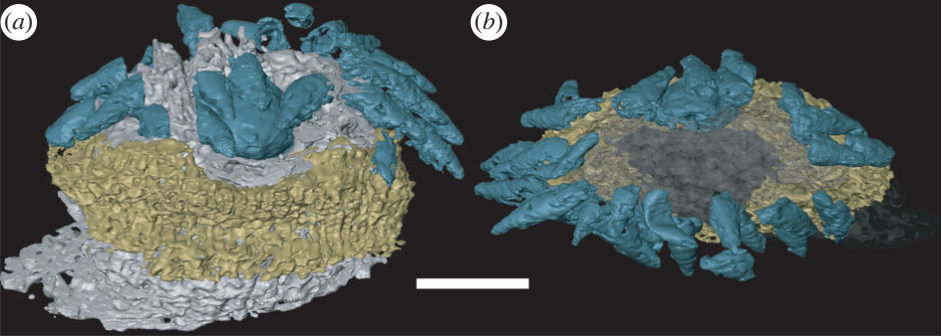
Three-dimensional model of the anterior structure and scalids, together with a three-dimensional model of pharynx to illustrate their relative position (GMPKU3139). (*a*) Dorsal view and (*b*) anterior view of the head of the specimen. Scale bar represents 60 µm (*a,b*).

The anterior structure occurs just aboral of the mouth and is positioned beneath the scalids (figure 5). Segmentation of the anterior structure using computed tomography reveals an oval ring-like structure that is approximately bilateral (figure 3), symmetrical about a plane that runs through the robust dorsal region and the thinner ventral region. The aboral margin is extended into a flange that expands radially (figure 3*c–f*). However, the thinner portion of the ring coincides with a region in which introvert scalids are compressed into the body, deforming this internal structure which would originally have been a complete and continuous ring. The lateral extremities of the oval ring exhibit a pair of symmetrical aboral projections, approximately 50 µm in length (figure 3*e,f*).

The posterior structure is more lightly mineralized than its anterior counterpart, but it otherwise exhibits the same mineral fabric (figure 4). Its morphology reflects the bilateral organization of the posterior end of the body, which it closely resembles. Its strongly compressed ovate cross-sectional profile does not form a complete ring, incomplete ventrally (based on orientation defined by the terminal spines). The structure circumscribes an ovate space that extends adorally along the body axis.

## 4. Discussion

The anatomy of the new specimens is clearly compatible with the existing concept of *Markuelia hunanensis* (text-fig. 1 in Dong *et al*. [1]). The new anatomical insights are afforded by the internal structures preserved with a distinct fabric of calcium phosphate mineralization. We interpret these as mineral replicates of original biological structures because of their distinct mineralization fabric and coherent overall morphology, as revealed by computed tomographic reconstruction. In particular, the morphology of the circumoral structure is distinct from surrounding and enveloping structures, hence, its distinctive morphology cannot merely be an internal mould. The mineralization fabric does not necessarily reflect the original biological structure, though it could reflect replication of the structure of a decay stage. Conservatively, we do not extend our identification of biological structure beyond gross morphology. The structure has undergone some deformation, perhaps as a result of decay-induced collapse; there is certainly evidence for compression of the overlying introvert (figure 2*b*), consistent with translational compression.

Topologically, in comparison with scalidophorans, the anterior structure is not compatible with the retractor musculature associated with the introvert which in priapulids, loriciferans, kinorhynchs and nematomorphs is arranged longitudinally [21–25]. Rather, this region of the anatomy of scalidophorans is occupied by the ring-shaped brain that gives its name to the broader paraphyletic group Cycloneuralia which is composed of scalidophorans plus nematoids (nematodes and nematomorphs) [26,27]. The ring-shaped brain is considered a consequence of the radial arrangement of sensory structures, including the scalids of scalidophorans, around the terminal mouth (figure 5), contrasting with the ventral mouth of panarthropods, the non-radial arrangement of their sensory organs and dorsal location of their brains [28]. As well as location, the size and shape of cycloneuralian brains [28] (including both soma, or neuron, and neuropil) are compatible with the anterior internal ring-shaped structure in *Markuelia* and we interpret it as such. The alternative interpretation that the fossilized features represent circular muscle [17] is incompatible with scalidophoran and cycloneuralian-grade anatomy.

The presence of a circular brain in *Markuelia* is an expectation of its scalidophoran affinity; setting aside the improbability of its fossilization, it would be surprising were it to exhibit a different morphology. The nature of the paired aboral projections may be more surprising since, accepting a brain interpretation of the ring-shaped anterior structure, their most likely interpretation is as the proximal end of longitudinal nerve cords. However, ecdysozoan nerve cords vary principally in terms of whether the ventral nerve cord is unpaired, bifid or paired [29]; nematodes and some nematomorphs also possess a dorsal nerve cord, but this is limited to processes extending from cell bodies in the main ventral unpaired nerve cord [30]. Thus, the paired aboral projections (figure 3*e,f*) may represent the origins of a main and subsidiary nerve cord.

The longitudinally extending structure associated with the posterior spines (figure 4) is mostly plausibly interpreted as musculature given its intimate association with the three pairs of caudal spines. These find no comparison to caudal structures in extant cycloneuralians, except perhaps the penile spines of kinorhynchs [31], but then only on topological grounds.

Nervous tissues have not previously been described from fossil scalidophorans and, indeed, taphonomy experiments based on priapulids indicate that these are among the first to decay, perhaps making these tissues the least likely to be fossilized under normal conditions [32]. However, there have now been numerous reports of fossilization of aspects of the gross anatomy of ecdysozoan nervous systems from Cambrian Burgess Shale-type Lagerstätte [33–41], demonstrating that factors other than decay resistance contribute to fossilization potential, including biochemistry and environmental context [42–44]. Necessarily, given the style of preservation, these are all effectively collapsed into two dimensions. Thus, with the exception of simple endocasts of boney vertebrate braincases, this is the first record of three-dimensional preservation of nervous system characters, suggesting that preservation of nervous tissue may not be limited to Burgess Shale-type deposits. Orsten-style preservation, which characterizes the preservation of *Markuelia*, is notorious for exquisite three-dimensional preservation of cuticularized tissues, but the lack of preservation of internal organs [45]. However, exceptions to this rule are known [46,47] and it remains possible that tomographic analysis of Orsten-style fossils (especially fossil embryos which have much more potential to preserve the soft tissues such as nerve, blood vessel and heart than that of larvae [48]) might be repaid by further insights into the internal anatomy and even the neural anatomy of the early ecdysozoans preserved in these deposits. Orsten-style fossils that exhibit filament-like internal mineral fabrics, preserved in diagenetic calcium phosphate, are more likely to preserve internal organs than specimens that are otherwise hollow or filled with euhedral-calcite void-filling cements.

In summary, we report the recovery of a large collection of embryos of the scalidophoran *Markuelia hunanensis* from the type locality within the Furongian (upper Cambrian) of Wangcun, Hunan, South China. These specimens conform to existing descriptions, but tomographic analyses reveal preserved internal anatomy in one specimen, limited to the anterior and posterior anatomical regions. These structures are interpreted as musculature associated with the terminal spine pairs and a ring-shaped brain. While these features might be anticipated of the ‘cycloneuralian’ scalidophoran affinity of *Markuelia*, they nevertheless extend records of the fossilization of nervous system anatomy from Burgess Shale-type Lagerstätten in which preservation is largely two-dimensional, to Orsten-type Lagerstätten in which preservation is three-dimensional.

## Data Availability

The underpinning tomographic data are available from the University of Bristol Research Data Storage Facility (data.bris) at https://doi.org/10.5523/bris.za3bxfhxpyct2qsy8ceeriksh [49].
